# Filamin C is Essential for mammalian myocardial integrity

**DOI:** 10.1371/journal.pgen.1010630

**Published:** 2023-01-27

**Authors:** Tongbin Wu, Yujun Xu, Lunfeng Zhang, Zhengyu Liang, Xiaohai Zhou, Sylvia M. Evans, Ju Chen

**Affiliations:** 1 Department of Medicine, University of California San Diego, La Jolla, California, United States of America; 2 Department of Pharmacology, Skaggs School of Pharmacy and Pharmaceutical Sciences, University of California San Diego, La Jolla, California, United States of America; 3 Department of Cellular and Molecular Medicine, University of California San Diego, La Jolla, California, United States of America; Indiana University Purdue University at Indianapolis, UNITED STATES

## Abstract

*FLNC*, encoding filamin C, is one of the most mutated genes in dilated and hypertrophic cardiomyopathy. However, the precise role of filamin C in mammalian heart remains unclear. In this study, we demonstrated *Flnc* global (*Flnc*^*gKO*^) and cardiomyocyte-specific knockout (*Flnc*^*cKO*^) mice died in utero from severely ruptured ventricular myocardium, indicating filamin C is required to maintain the structural integrity of myocardium in the mammalian heart. Contrary to the common belief that filamin C acts as an integrin inactivator, we observed attenuated activation of β1 integrin specifically in the myocardium of *Flnc*^*gKO*^ mice. Although deleting β1 integrin from cardiomyocytes did not recapitulate the heart rupture phenotype in *Flnc* knockout mice, deleting both β1 integrin and filamin C from cardiomyocytes resulted in much more severe heart ruptures than deleting filamin C alone. Our results demonstrated that filamin C works in concert with β1 integrin to maintain the structural integrity of myocardium during mammalian heart development.

## Author summary

The precise role of filamin C in mammalian heart development had not been determined, in part due to the lack of cardiac phenotypes in previously described *Flnc* knockout mice, which still had truncated filamin C expressed in the heart. In this study, we analyzed a true *Flnc* knockout mouse line, in which filamin C protein was completely ablated. *Flnc* knockout mice developed massive ruptures in their myocardium but not in the endocardium, suggesting filamin C is essential for the structural integrity of myocardium. On the other hand, we did not find overt abnormalities of sarcomeric structure in cardiomyocytes of *Flnc* knockout mice, indicating that filamin C is likely not required for sarcomeric assembly as previously observed in *FLNC* null iPSC-CMs. Moreover, contrary to the dogma that filamins are integrin inactivators, we found that filamin C plays an unexpected role in integrin activation and works in concert with β1 integrin to ensure the structural integrity of the myocardium.

## Introduction

Cardiomyopathy is one of the leading causes of morbidity and mortality around the world [1]. Genetic causes, including detrimental deletions, insertions, nonsense or missense mutations identified in nearly 100 genes, account for diverse forms of hypertrophic, dilated, restrictive, and arrhythmogenic cardiomyopathy [2, 3]. One of the most mutated genes is *FLNC* (encoding filamin C), which has 77 variants identified among dilated cardiomyopathy (DCM) and 57 variants in hypertrophic cardiomyopathy (HCM) patients, many of which are pathogenic [4].

Filamins (FLNA, FLNB, FLNC) are large actin-binding and -crosslinking dimeric proteins, with each subunit ranging from 240 to 280 kDa [5]. Filamin C (FLNC) is predominantly expressed in striated muscle tissues [6], and is localized to the Z-disc [7], intercalated disc (ICD) [8], and costamere [6]. Filamin C contains an N-terminal actin-binding domain (ABD) and 24 C-terminal immunoglobulin (Ig)-like domains [5], which are responsible for protein dimerization and interacting with myotilin and FATZ-1 at Z-discs [9, 10]. C-terminal Ig-like domains also interact with β1 integrin [11] and sarcoglycans [6] at the costamere, a structural and functional component that bridges and strengthens the connection of the Z-discs to the sarcolemma [12]. Thus, filamin C is proposed to serve as a link between myofibrils and sarcolemma [7, 13, 14]. *In vitro* studies have demonstrated filamins inactivate integrin by competing with talin for binding to the cytoplasmic domain of the integrin β subunit [15]. However, the functional consequences of loss of filamins, especially filamin C, on integrin activation and its potential role in filamin C-related cardiomyopathy has not been explored *in vivo*.

Several studies sought to elucidate the function of filamin C in heart. A nonsense mutation identified in the teleost fish medaka causes myocardial rupture in heart ventricles, suggesting that filamin C is involved in maintenance of structural integrity of cardiac muscle [16]. Ablating filamin C in human induced pluripotent stem cell–derived cardiomyocytes (hiPSC-CMs) led to sarcomere disarray [17]. Surprisingly, mice with homozygous deletion of the last 8 exons of *Flnc* did not show overt cardiac phenotypes [18]. However, these *Flnc* knockout mice still expressed a truncated form of filamin C protein in the heart [18]. Thus, a *bona fide Flnc* knockout mouse model is required to study the precise role of filamin C in mammalian heart.

To this end, we generated a floxed *Flnc* mouse line [19] and analyzed *Flnc* global knockout (*Flnc*^*gKO*^) and *Flnc* cardiomyocyte-specific knockout (*Flnc*^*cKO*^) mice. Both *Flnc*^*gKO*^ and *Flnc*^*cKO*^ mice died before embryonic day (E) 11.5 from severely ruptured ventricular myocardium, indicating filamin C is required to maintain the structural integrity of myocardium in mammalian heart. By immunofluorescence analyses, we found downregulation of key extracellular matrix (ECM) proteins which might partially explain the heart rupture phenotype. Surprisingly, we did not observe obvious sarcomere disarray in cardiomyocytes of *Flnc*^*gKO*^ mice, suggesting that filamin C is not required for sarcomere assembly *in vivo*. Interestingly, we observed attenuated activation of β1 integrin specifically in myocardium of *Flnc*^*gKO*^ mice. However, deleting β1 integrin from cardiomyocytes did not recapitulate the heart rupture phenotype in *Flnc* knockout mice, whereas deleting both β1 integrin and filamin C from cardiomyocytes resulted in larger heart ruptures. Our results demonstrated that filamin C works in concert with β1 integrin to maintain the structural integrity of myocardium during mammalian heart development.

## Results

### Filamin C is essential for mammalian heart development

**Fig 1:**
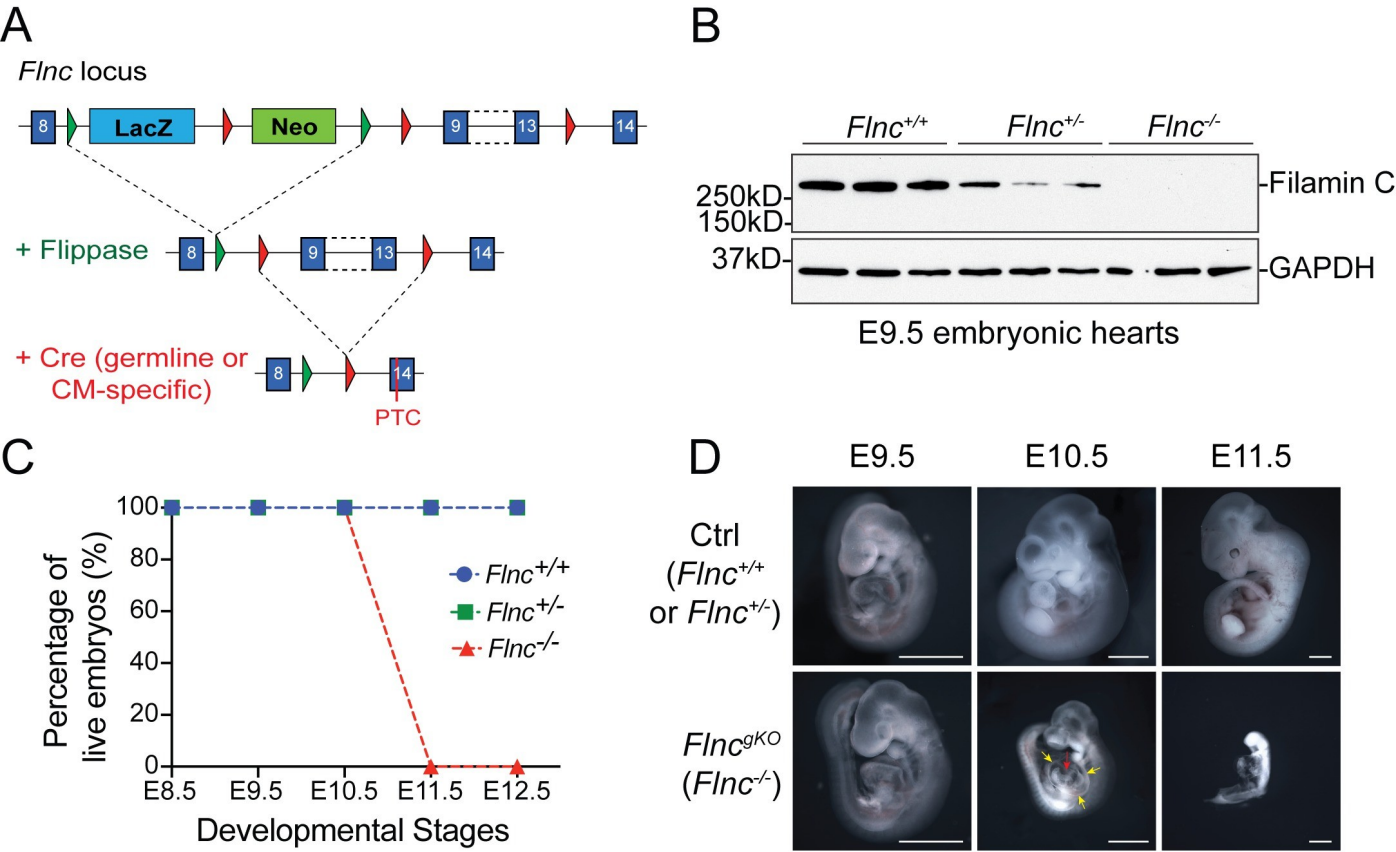
Filamin C is essential for mammalian heart development. (**A**) Targeting strategy for generating *Flnc* knockout mice. Global *Flnc* knockout mice were generated by crossing *Flnc* floxed mice (*Flnc*^*fl/fl*^) with *Sox2*^*Cre*^ mice, while cardiomyocyte-specific *Flnc* knockout mice were generated by crossing *Flnc*^*fl/fl*^ mice with *Xmlc2*^*Cre*^ mice or *cTnT*^*Cre*^ mice. After the deletion of exon 9–13 of the *Flnc* gene by Cre recombinase, a premature termination codon (PTC) will be introduced to exon 14 and trigger non-sense mediated mRNA decay (NMD) of *Flnc* mRNA. (**B**) Western blot confirms the complete depletion of filamin C protein in *Flnc* global knockout mice (*Flnc*^*-/-*^ or *Flnc*^*gKO*^). GAPDH is used as a loading control. (**C**) Percentage of live wild-type (*Flnc*^*+/+*^), heterozygous (*Flnc*^*+/-*^) and knockout (*Flnc*^*-/-*^) embryos from E8.5 to E12.5. Exact number of each genotype and each developmental stage: E8.5, 4:9:4 (*Flnc*^*+/+*^: *Flnc*^*+/-*^: *Flnc*^*-/-*^); E9.5, 16:32:15; E10.5, 13:25:11; E11.5, 4:7:4*; E12.5, 4:6:0. *: dead/under resorption. (**D**) Wholemount images of control and *Flnc* global knockout (*Flnc*^*gKO*^) embryos at E9.5, E10.5 and E11.5. Yellow arrows indicate pericardial effusion; Red arrow indicates heart rupture; Scale bar, 1 mm.

By *in situ* hybridization, we demonstrated that *Flnc* was specifically expressed in heart and somites from embryonic day (E) 9.5 to 11.5 (S1A Fig). The expression pattern of *Flnc* is consistent with the observation that heart and skeletal muscle are most affected in patients with mutations in *FLNC* [4, 20]. However, previously described *Flnc* knockout mice only had defects in skeletal muscles but not in heart, probably owing to the hypomorphic nature of the mutant *Flnc* allele in that study [18]. Thus, a *bona fide Flnc* knockout mouse model is required to fully understand roles of filamin C in heart. To this end, we generated global *Flnc* knockout mice (*Flnc*^*-/-*^ or *Flnc*^*gKO*^) by crossing *Flnc*^*fl/fl*^ [19] mice with *Sox2*^*Cre*^ mice [21] (Fig 1A). Western blot and immunofluorescence analyses confirmed that filamin C protein was completely absent in *Flnc*^*gKO*^ mice (Figs 1B and S1B).

In stark contrast to *Flnc* hypomorphic mice [18], *Flnc*^*gKO*^ mice developed pericardial effusion, a hallmark of insufficient cardiac function [22], at E10.5, and died before E11.5 (Fig 1C–1D). To determine whether the embryonic lethality of *Flnc*^*gKO*^ mice was due to loss of cell-autonomous function of filamin C in cardiomyocytes, we generated cardiomyocyte-specific *Flnc* knockout mice by crossing *Flnc*^*fl/fl*^ mice with *Xmlc2*^*Cre*^ mice [23] or *cTnT*^*Cre*^ mice [24]. Both cardiomyocyte-specific *Flnc* knockout mouse lines exhibited identical phenotypes to those of *Flnc*^*gKO*^ mice, and died before E11.5 (S1C–S1D Fig), indicating filamin C is essential in developing cardiomyocytes.

### Filamin C maintains the integrity of the myocardial wall

**Fig 2:**
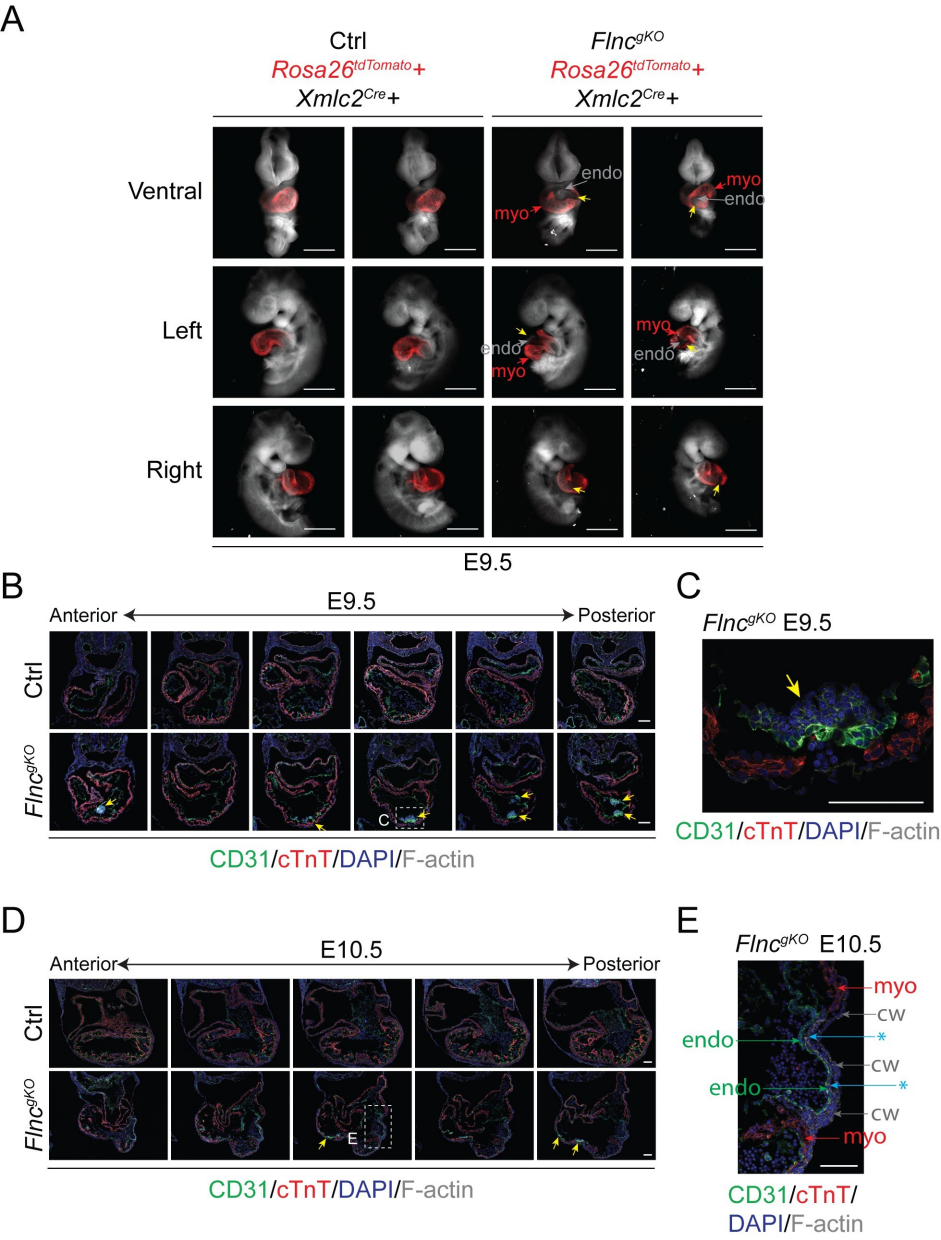
Filamin C maintains the integrity of myocardial wall. (**A**) Wholemount brightfield/fluorescence merged images of control and *Flnc*^*gKO*^ embryos with red fluorescence myocardium indicator at E9.5. Yellow arrows indicate heart ruptures. Endo, endocardium; Myo, myocardium. Scale bar, 0.5 mm. (**B** and **C**) Representative immunofluorescence (IF) images of control and *Flnc*^*gKO*^ hearts at E9.5. Antibodies used for IF are indicated. Yellow arrows indicate CD31-positive thrombi. Scale bar, 0.1 mm. (**D** and **E**) Representative immunofluorescence (IF) images of control and *Flnc*^*gKO*^ hearts at E10.5. Antibodies used for IF are indicated. Yellow arrows indicate heart ruptures. CW, chest wall; Asterisk, chest wall overgrowth. Scale bar, 0.1 mm.

Upon close examination of E10.5 *Flnc*^*gKO*^ mouse hearts, we observed ruptures in the ventricular wall (Fig 1D). To determine the exact location of the ruptures, we intercrossed *Flnc*^*+/-*^ mice with *Rosa26*^*tdTomato*^/*Xmlc2*^*Cre*^ to label cardiomyocytes with tdTomato fluorescence [25, 26]. Results showed that *Flnc*^*gKO*^ mice had multiple ruptures in myocardium but not in endocardium (Fig 2A). Interestingly, the location of rupture sites varied between individual *Flnc*^*gKO*^ mice (Fig 2A).

To better characterize the heart rupture phenotype, we sectioned E8.5-E10.5 *Flnc*^*gKO*^ hearts and performed immunofluorescence (IF) staining. We did not find any ruptures in the myocardial wall of E8.5 *Flnc*^*gKO*^ hearts (S2A Fig), suggesting that heart rupturing occurred after E8.5. In accordance with our observations in wholemount hearts (Fig 2A), the myocardium of E9.5 *Flnc*^*gKO*^ hearts had multiple rupture sites while the endocardium remained intact (Fig 2B). Interestingly, we found CD31-positive thrombi accumulated at the rupture sites in E9.5-E10.5 *Flnc*^*gKO*^ hearts (Fig 2B–2D), and chest wall tissues had overgrown large rupture sites at E10.5 (Fig 2D–2E).

To investigate whether the heart rupturing was caused by cardiomyocyte hypoplasia resulting from decreased cardiomyocyte proliferation and/or increased cardiomyocyte apoptosis, we measured cardiomyocyte proliferation and apoptosis rates in E8.5 to E10.5 *Flnc*^*gKO*^ hearts and littermate controls. Although cardiomyocyte proliferation was markedly reduced and cardiomyocyte apoptosis was increased in E10.5 *Flnc*^*gKO*^ hearts compared with controls, both parameters were indistinguishable between *Flnc*^*gKO*^ and controls from E8.5 to E9.5 (S2B–S2C Fig). Because the heart rupture phenotype was already evident in E9.5 *Flnc*^*gKO*^ hearts, these findings indicated that heart rupturing was not caused by cardiomyocyte hypoplasia.

As filamin C is thought to play a role in sarcomere assembly in iPSC-CMs *in vitro* [17], we examined the overall sarcomere structure in E9.5 *Flnc*^*gKO*^ cardiomyocytes by IF using antibodies against α-actinin (Z line) or myomesin (M line). However, we did not find any obvious sarcomere disarray in *Flnc*^*gKO*^ hearts (S2D Fig), indicating filamin C is dispensable for sarcomere assembly *in vivo*.

### Wound healing and blood coagulation pathways were activated in *Flnc*^*gKO*^

**Fig 3:**
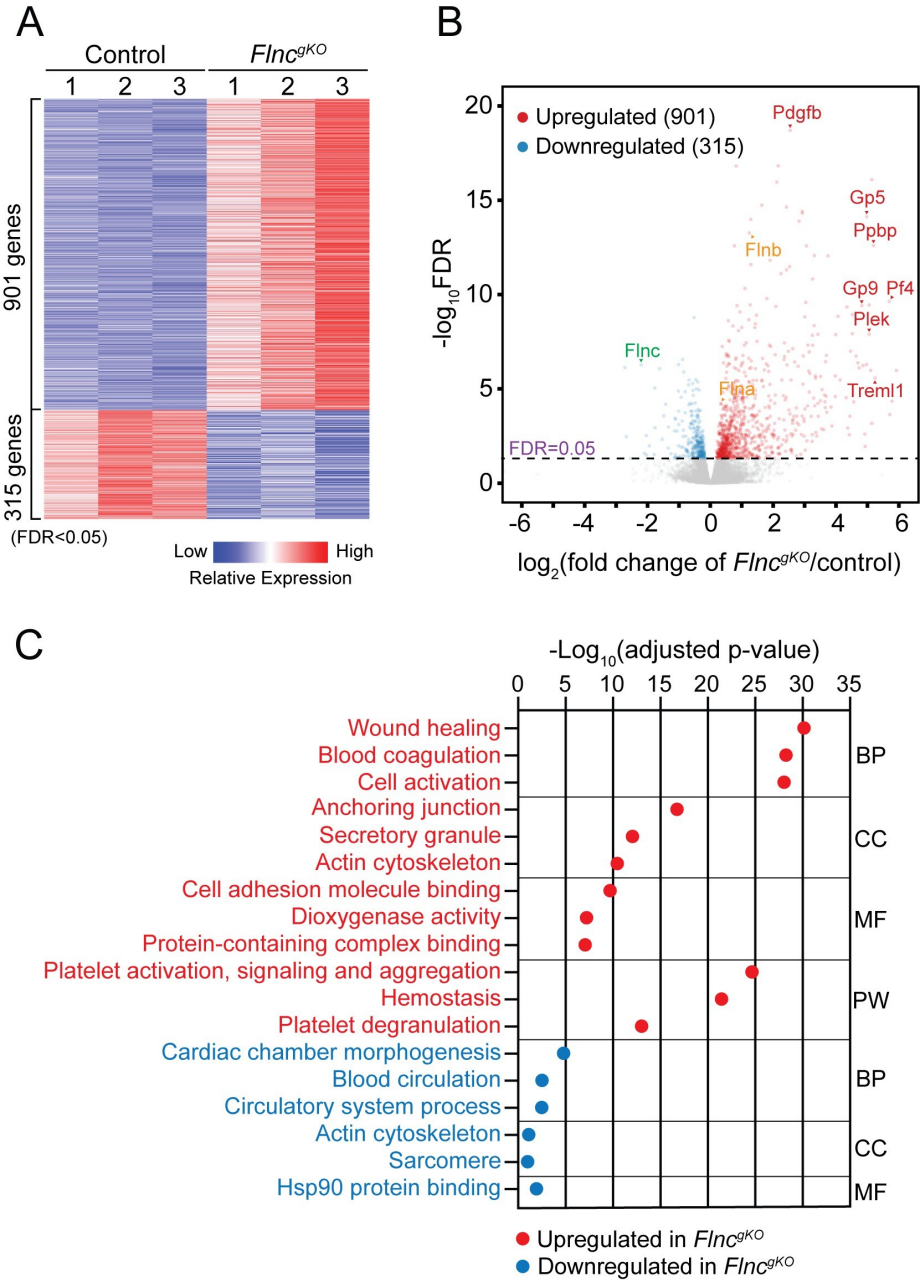
Wound healing and blood coagulation pathways were activated in *Flnc*^*gKO*^. (**A**) Heatmap showing gene expression changes in E9.5 control and *Flnc*^*gKO*^ hearts (n = 3 per group). (**B**) Volcano plot of differentially expressed genes (DEGs) [false discovery rate (FDR) < 0.05)] in E9.5 *Flnc*^*gKO*^ hearts compared with littermate controls. Notable DEGs are indicated. (**C**) Gene ontology analysis of differentially expressed genes of *Flnc*^*gKO*^ hearts compared with littermate controls at E9.5. BP, biological process; CC, cellular component; MF, molecular function; PW, KEGG pathway.

To assess transcriptomic changes in *Flnc*^*gKO*^ mice, we extracted RNA from E9.5 *Flnc*^*gKO*^ and littermate control hearts and performed RNA sequencing (RNA-seq). Using false discovery rate (FDR) < 0.05, we identified 901 significantly upregulated and 315 significantly downregulated differentially expressed genes (DEGs) in *Flnc*^*gKO*^ hearts (Fig 3A–3B and S1 Table). Among the most downregulated DEGs was *Flnc*, indicating our RNA-seq faithfully reflected gene expression changes between *Flnc*^*gKO*^ and controls (Fig 3B). Gene ontology analysis revealed downregulated DEGs enriched in molecular pathways related to cardiac chamber morphogenesis and function (Fig 3C), which might contribute to the cardiac phenotypes observed in *Flnc*^*gKO*^ mice.

On the other hand, we found that genes involved in blood coagulation, including *Pdgfb* [27], *Ppbp* [28] and *Gp5* [29] (Fig 3B) were dramatically upregulated in *Flnc*^*gKO*^ hearts. Gene ontology analysis demonstrated upregulated DEGs mostly enriched in blood coagulation and wound healing processes (Fig 3C), in agreement with the formation of thrombi at rupture sites in *Flnc*^*gKO*^ hearts (Fig 2B–2D). In addition, we found compensatory upregulation of *Flna* and *Flnb* in *Flnc*^*gKO*^ hearts (Fig 3B).

### Extracellular matrix proteins are downregulated in the myocardium of *Flnc*^*gKO*^

**Fig 4:**
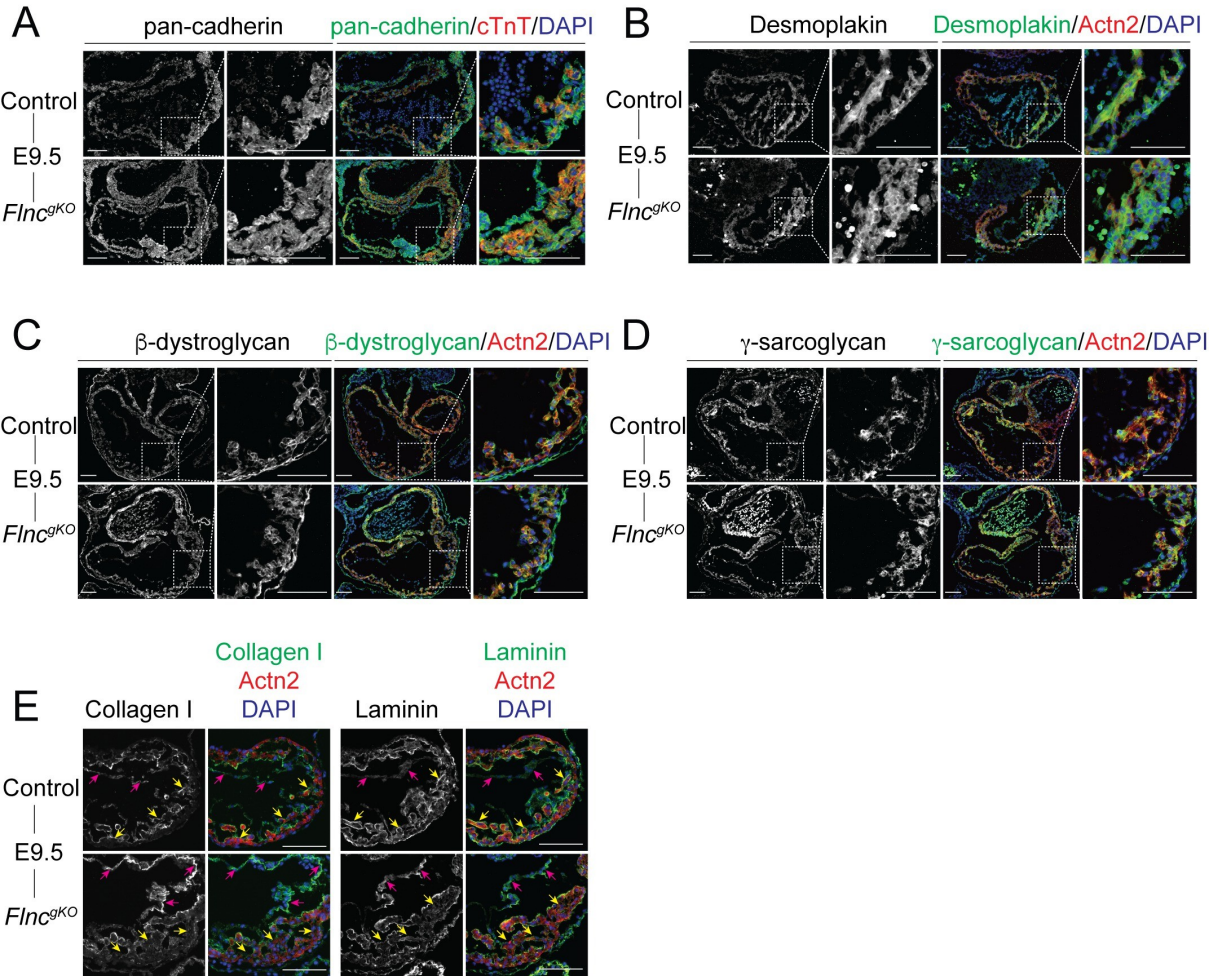
Extracellular matrix proteins are downregulated in the myocardium of *Flnc*^*gKO*^. (**A-D**) Representative immunofluorescence (IF) images of control and *Flnc*^*gKO*^ hearts at E9.5 using antibodies against α-actinin (actn2) or cardiac troponin T (cTnT), and pan-cadherin (**A**), desmoplakin (**B**), β-dystroglycan (**C**) or γ-sarcoglycan (**D**). Scale bar, 0.1 mm. (**E**) Representative collagen I and laminin immunofluorescence images of control and *Flnc*^*gKO*^ hearts at E9.5. yellow arrows: myocardium; magenta arrows: endocardium. Scale bar, 0.1 mm.

As cell-cell junctions of cardiomyocytes are critical for structural integrity of the heart [30], we examined expression and localization of key cell-cell junction proteins, including cadherins and desmoplakin, in E9.5 *Flnc*^*gKO*^ hearts (Fig 4A–4B). However, we found their expression and localization were comparable between *Flnc*^*gKO*^ and controls, indicating the heart rupture phenotype was not caused by diminished expression or mislocalization of cell-cell junction proteins.

Because filamin C is localized to costameres and interacts with the dystrophin-associated glycoprotein complex (DGC) [6], we then examined expression and localization of the DGC proteins β-dystroglycan and γ-sarcoglycan and found they were not reduced or mislocalized in E9.5 *Flnc*^*gKO*^ hearts (Fig 4C–4D), indicating the heart rupture phenotype in *Flnc*^*gKO*^ hearts was not caused by dysregulation of the DGC complex.

We previously reported extracellular matrix (ECM) disorganization caused heart rupture phenotypes in kindlin-2 cardiomyocyte-specific knockout mice [31]. To determine whether filamin C is required for the proper organization of ECM, we performed IF of ECM proteins collagen I and laminin on E9.5 *Flnc*^*gKO*^ hearts. Although the expression of collagen I and laminin were upregulated in endocardium, they were markedly downregulated in myocardium of *Flnc*^*gKO*^ hearts (Fig 4E), which may partially account for the myocardial rupture phenotype in *Flnc*^*gKO*^ mice.

### Both filamin C and β1 integrin are required to maintain the structural integrity of myocardium

**Fig 5:**
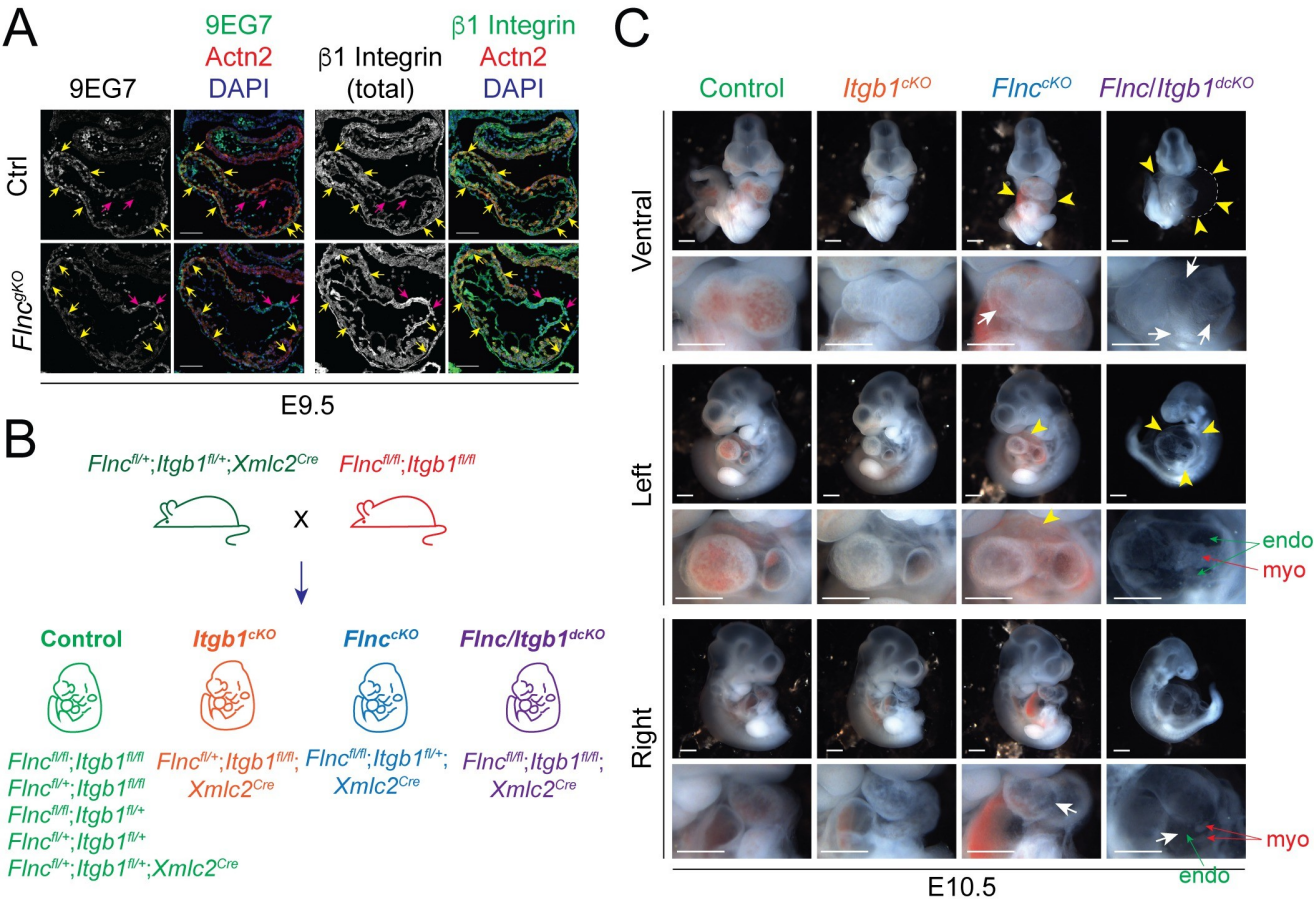
Both filamin C and β1 integrin is required to maintain the structural integrity of myocardium. (**A**) Representative activated (9EG7) and total β1 integrin immunofluorescence images of control and *Flnc*^*gKO*^ hearts at E9.5. yellow arrows: myocardium; magenta arrows: endocardium. Scale bar, 0.1 mm. (**B**) The strategy for generating cardiomyocyte-specific *Itgb1* knockout mice (*Itgb1*^*cKO*^), cardiomyocyte-specific *Flnc* knockout mice (*Flnc*^*cKO*^) and cardiomyocyte-specific *Flnc/Itgb1* double knockout mice (*Flnc/Itgb1*^*dcKO*^). (**C**) Wholemount images of control, *Itgb1*^*cKO*^, *Flnc*^*cKO*^ and *Flnc/Itgb1*^*dcKO*^ embryos at E10.5. Yellow arrowheads indicate pericardial effusion; White arrows indicate heart ruptures. Scale bar, 0.5 mm.

Filamins are known integrin inactivators and abnormal activation of β1 integrin can lead to impaired cell proliferation, differentiation and migration [15]. To determine whether β1 integrin, a dominant integrin β isoform in cardiomyocytes [31], was ectopically activated in *Flnc*^*gKO*^ mice, we performed IF using an antibody (9EG7) [32] against the activated ligand-bound conformation of β1 integrin and an antibody against total β1 integrin. Surprisingly, we found that activated β1 integrin was reduced in the myocardium of *Flnc*^*gKO*^ hearts without changes in the total β1 integrin expression (Fig 5A). In contrast, both activated and total β1 integrin were upregulated in regions of endocardium proximal to rupture sites of *Flnc*^*gKO*^ hearts (Fig 5A).

To assess whether decreased activation of β1 integrin in cardiomyocytes contributed to the heart rupture phenotype observed in *Flnc*^*gKO*^ mice, we generated cardiomyocyte-specific *Itgb1* (encoding β1 integrin) knockout mice using *Xmlc2*^*Cre*^ (*Itgb1*^*cKO*^) and compared them with cardiomyocyte-specific *Flnc* knockout mice (*Flnc*^*cKO*^) (Fig 5B). Consistent with observations in *Flnc*^*gKO*^ mice, *Flnc*^*cKO*^ mice had obvious ruptures in their myocardium at E10.5 (Fig 5C). Although we previously reported that *Itgb1*^*cKO*^ develop heart ruptures at E14.5 [31]. *Itgb1*^*cKO*^ hearts did not show rupture at E10.5 (Fig 5C). These findings indicated that decreased β1 integrin activation in *Flnc*^*gKO*^ mice may only partially account for the myocardial rupture phenotype.

To further explore the genetic interaction of *Flnc* and *Itgb1*, and its contribution to myocardial wall integrity, we generated cardiomyocyte-specific *Flnc*/*Itgb1* double knockout mice (*Flnc*/*Itgb1*^*dcKO*^) (Fig 5B). Strikingly, *Flnc*/*Itgb1*^*dcKO*^ mice had much more severe heart rupturing which led to myocardial disintegration (Fig 5C), compared with *Flnc*^*cKO*^ mice. Consequently, the endocardium of *Flnc*/*Itgb1*^*dcKO*^ mice became inflated, presumably due to the lack of mechanical support from the myocardium (Fig 5C). *Flnc*/*Itgb1*^*dcKO*^ mice also had larger pericardial effusions and more pronounced overall growth retardation (Fig 5C). Taken together, our findings suggest that filamin C works in concert with β1 integrin to maintain the structural integrity of myocardium during mammalian heart development.

## Discussion

In this study, we demonstrated that filamin C played an essential role in maintaining the structural integrity of myocardium, as *Flnc*^*gKO*^ mice had severely ruptured ventricular myocardium but intact endocardium. Interestingly, CD31-positive thrombi and chest wall overgrowth were observed at the rupture sites, and β1 integrin and ECM proteins were upregulated in the endocardium. These phenomena are likely compensatory mechanisms to prevent complete heart rupturing. However, *Flnc*^*gKO*^ mice did not survive past E11.5, indicating that filamin C is essential for heart development and embryonic survival. Mechanistically, although several cell junction and dystrophin-associated glycoprotein complex (DGC) proteins were unchanged, key extracellular matrix (ECM) proteins were downregulated in myocardium of *Flnc*^*gKO*^ mice which may partially explain the heart rupture phenotype, reminiscent of our findings in kindlin-2 knockout mice [31]. Contrary to the belief that filamin C functions as an integrin inactivator, we observed attenuated activation of β1 integrin specifically in the myocardium of *Flnc*^*gKO*^ mice. To further investigate whether downregulation of activated β1 integrin was key to cardiac phenotypes in *Flnc*^*gKO*^ mice, we generated β1 integrin cardiomyocyte-specific knockout mice (*Itgb1*^*cKO*^). However, *Itgb1*^*cKO*^ mice did not recapitulate the early heart rupture phenotype observed in *Flnc* knockout mice, whereas deleting β1 integrin and filamin C simultaneously from cardiomyocytes resulted in much more severe heart ruptures. Our results suggest filamin C works in concert with β1 integrin to maintain the structural integrity of myocardium during mammalian heart development.

*FLNC is* among the most mutated genes in dilated cardiomyopathy (DCM) and hypertrophic cardiomyopathy (HCM) patients [4], underscoring the essential role of filamin C in cardiac development and function. However, previously described *Flnc* knockout mice with homozygous deletion of the last 8 exons of *Flnc* only had defects in skeletal muscle but not in cardiac muscle [18]. Further studies revealed that these mice still expressed a truncated form of filamin C protein lacking the last four immunoglobulin (Ig)-like repeats and the hinge 2 region [18]. While the truncated filamin C protein was expressed at a lower level than wildtype, the reduction in *Flnc* mRNA levels was less pronounced in heart than in limb muscle [18], which may explain why there are phenotypes in skeletal muscles but not in heart of *Flnc* knockout mice. These observations also suggested that the N-terminal actin-binding domain and 19 Ig-like repeats (~82% of wild-type protein), even at much lower levels than that of wild-type FLNC proteins, are sufficient for filamin C to function normally in heart. Thus, the hypomorphic nature of the mutant *Flnc* allele renders it unsuitable for studying the function of filamin C in the heart. To address this problem, we generated *Flnc* knockout mice by deleting exon 9–13 of the *Flnc* gene, which introduced a premature termination codon (PTC) within exon 14 and subjected *Flnc* mRNA to nonsense-mediated mRNA decay (NMD). In line with this, *Flnc* mRNA levels were drastically downregulated in *Flnc* global knockout mice according to our RNA-seq data (Fig 3B and S1 Table). Although a small amount of N-terminal truncated protein (not recognizable by our filamin C antibodies that were raised against C-terminal regions of filamin C protein) could be generated, the truncated protein is unlikely to be functional as it only includes the N-terminal actin-binding domain and two Ig-like domains (486 amino acids, ~17% of wild-type protein).

In a recent report, filamin C was ablated in *in vitro* cultured human induced pluripotent stem cell–derived cardiomyocytes (*FLNC*^*−/−*^ hiPSC-CMs), which exhibited defects in sarcomere assembly and decreased thin filament gene expression, suggesting that filamin C plays a role in sarcomere assembly and thin filament gene expression [17]. To determine whether filamin C possesses similar functions *in vivo*, we examined overall sarcomere structure in E9.5 *Flnc*^*gKO*^ cardiomyocytes by immunofluorescence analyses. However, we did not observe any sarcomere disarray as seen in *FLNC*^*−/−*^ hiPSC-CMs. In addition, our RNA-seq analysis revealed very modest downregulation of thin filament genes including *Lmod2* (Log_2_FC = -0.33), *Tnni3* (Log_2_FC = -0.30) and *Synpo2* (Log_2_FC = -0.31) (S1 Table), which is in stark contrast to the dramatic downregulation of thin filament genes in *FLNC*^*−/−*^ hiPSC-CMs [17]. Our findings suggest that filamin C is dispensable in sarcomere assembly and has minimal impact on the expression of thin filament genes *in vivo*. It is worth noting that filamin A and filamin B were not upregulated in *FLNC*^*−/−*^ hiPSC-CMs according to our examination of the transcriptomics and proteomics data from that study [17]. Thus, upregulation of filamin A and filamin B, or lack thereof, could explain why there are no defects of sarcomere assembly in cardiomyocytes of *Flnc*^*gKO*^ mice but sarcomere disarray in *FLNC*^*−/−*^ hiPSC-CMs. Future studies, i.e., ablating all three filamins from cardiomyocytes *in vivo*, might be necessary to elucidate roles of filamins in sarcomere assembly.

Filamin C interacts with β1 integrin [11] and sarcoglycans [6] at the costamere to serve as a link between myofibrils and sarcolemma. Our discovery of myocardial wall ruptures in *Flnc* knockout mice provided strong support for filamin C’s essential structural role in myocardium integrity. On the other hand, filamins are well-known integrin inactivators that function by competing with talin for binding to the cytoplasmic domain of the integrin β subunit [15], and abnormal activation of β1 integrin can lead to impaired cell proliferation, differentiation and migration [15]. To determine whether β1 integrin is ectopically activated in filamin C-ablated cardiomyocytes which could account for the observed cardiac phenotypes, we examined expression and localization of activated and total β1 integrin by immunofluorescence. Surprisingly, while total β1 integrin expression and localization were unchanged, activated β1 integrin was reduced in myocardium of *Flnc*^*gKO*^ hearts. To further investigate whether the attenuated activation of β1 integrin was key to cardiac phenotypes in *Flnc*^*gKO*^ mice, we generated cardiomyocyte-specific *Itgb1* knockout mice (*Itgb1*^*cKO*^) and compare them with cardiomyocyte-specific *Flnc* knockout mice (*Flnc*^*cKO*^). However, ablating β1 integrin in cardiomyocytes did not cause myocardial rupture at E10.5, a stage when *Flnc*^*cKO*^ mice already had severe rupturing in their myocardium. Considering that *Itgb1*^*cKO*^ indeed develop heart ruptures later at E14.5 [31], these findings indicated that attenuated β1 integrin activation alone may only partially account for myocardial ruptures in *Flnc*^*gKO*^ mice. Another possibility is that some residual β1 integrin proteins may still remain in E10.5 *Itgb1*^*cKO*^ mice due to their remarkably long half-life [33], and these remaining β1 integrin proteins can be normally activated in the presence of filamin C. If this is the case, simultaneously ablating filamin C and β1 integrin should recapitulate the phenotypes of *Flnc*^*cKO*^ mice. However, the *Flnc*/*Itgb1*^*dcKO*^ mice we generated had even more severe heart rupturing phenotypes than *Flnc*^*cKO*^ mice, suggesting that filamin C maintains the integrity of myocardium through both integrin-dependent and integrin-independent pathways. Future studies are needed to delineate detailed molecular mechanisms by which filamin C facilitates β1 integrin activation in cardiomyocytes.

## Methods

### Ethics statement

All animal procedures were performed in accordance with the National Institutes of Health Guide for the Care and Use of Laboratory Animals and approved by the Institutional Animal Care and Use Committee of the University of California San Diego with approved protocol # S01049.

### Mice

*Flnc* and *Itgb1* floxed mice were generated previously [19, 34]. Global *Flnc* knockout mice were generated by crossing *Flnc* floxed mice (*Flnc*^*fl/fl*^) with *Sox2*^*Cre*^ mice [21], while cardiomyocyte-specific *Flnc* knockout mice were generated by crossing *Flnc*^*fl/fl*^ mice with *Xmlc2*^*Cre*^ mice [23] or *cTnT*^*Cre*^ mice [24]. Cardiomyocyte-specific *Itgb1* knockout mice were generated by crossing *Itgb1*^*fl/fl*^ mice [34] with *Xmlc2*^*Cre*^ mice. Cardiomyocyte-specific *Flnc*/*Itgb1* double knockout mice were generated by crossing *Flnc*^*fl/fl*^ mice with *Itgb1*^*fl/fl*^ mice and subsequently with *Xmlc2*^*Cre*^ mice. Genotyping of mice was confirmed by polymerase chain reaction (PCR) analysis using embryonic yolk sac extracts using *Flnc* WT allele primers (forward: 5’- TGGAGGTTGTAGGATCCCAG-3’; reverse: 5’- ATGTTAGTAGTCAGGGAGAGGC-3’), *Flnc* KO allele primers (forward: 5’- GCCCTGTGAGCTCCATGTATC-3’; reverse: 5’- TCAATGTTCGTAAAATTGATTAACAAGC-3’), *Flnc* floxed primers (forward: 5’- TGGAGGTTGTAGGATCCCAG-3’; reverse: 5’- ATGTTAGTAGTCAGGGAGAGGC-3’), *Itgb1* floxed primers (forward: 5’-AAGACAGGGTTTCTCTGTGTAG-3’; reverse: 5’-TATGAGGCTCCTTGATTGGTCA-3’), Cre primers (forward: 5’- GTTCGCAAGAACCTGATGGACA-3’; reverse: 5’-CTAGAGCCTGTTTTGCACGTTC-3’), and Rosa26-tdTomato primers (WT-forward: 5’- AAGGGAGCTGCAGTGGAGTA-3’, WT-reverse: 5’- CCGAAAATCTGTGGGAAGTC-3’, tdTomato-forward: 5’- CTGTTCCTGTACGGCATGG-3’, tdTomato-reverse: 5’- GGCATTAAAGCAGCGTATCC-3’).

### Western blots

Western blots were performed as previously described [26, 35]. Briefly, embryonic mouse hearts were dissected and snap-frozen in liquid nitrogen. Total protein extracts were prepared by homogenization of hearts in RIPA buffer using a handheld pellet pestle (Sigma-Aldrich). Protein samples were separated on Bolt 4%-12% Bis-Tris gels (Life Technologies) and transferred to PVDF membrane (Bio-Rad). Membranes were then blocked and incubated with primary antibodies overnight at 4°C. Membranes were then washed with TBST and incubated with HRP-conjugated secondary antibodies and visualized using enhanced chemiluminescence (ECL) reagent (Bio-Rad) and captured by Bio-Rad ChemiDoc Imaging System. Catalog numbers for antibodies used in western blots in this study: filamin C, NBP1-89300 (Novus); GAPDH, sc-32233 (Santa Cruz Biotechnology).

### Immunofluorescence

Immunofluorescence was performed as previously described [26, 35]. Briefly, embryonic mouse hearts were dissected at various developmental stages and fixed in ice-cold PBS with 4% PFA overnight at 4°C. Fixed hearts were then saturated in sucrose gradient and embedded in OCT Tissue-Tek (Thermo Fisher Scientific) for cryosectioning. Sections were blocked with PBST and incubated with primary antibody solution overnight in a humidified chamber at 4°C. The next day, sections were washed with PBST and then incubated with secondary antibody solution for two hours at room temperature. After washing with PBST, sections were counterstained with DAPI and mounted in DAKO fluorescence mounting medium (Agilent). Images were captured using Olympus FluoView FV1000 Confocal Microscope or ECHO Revolve Microscope. Catalogue numbers or sources for antibodies used in immunofluorescence in this study: filamin C (gift from Dr. Jun Qin, Cleveland Clinic), CD31 (550274, BD), α-actinin (650931, Sigma-Aldrich), cardiac troponin T (MS-295-P1, Thermo Fisher), myomesin (B4, DSHB), desmoplakin (2722–5204, Bio-Rad), β-dystroglycan (MANDAG2, DSHB), γ-sarcoglycan (VP-G803, Vector Laboratories), β1 integrin (MAB1997, Millipore), active β1 integrin/9EG7 (553715, BD), collagen I (ab34710, Abcam), laminin (ab11575, Abcam), pan-cadherin (031M4854, Sigma), phosphor histone-H3 (06–570, Millipore), cleaved caspase 3 (9661S, Cell Signaling).

### RNA Sequencing

RNA sequencing (RNA-seq) was performed as previously described [26]. E9.5 embryonic hearts or isolated ventricles were homogenized in TRIzol (Invitrogen) and total RNA was isolated according to the manufacturer’s instructions. The concentration and quality of purified RNA was assessed by TapeStation (Agilent). cDNA libraries were prepared using an Illumina TruSeq stranded mRNA kit according to manufacturer’s instructions. Libraries were sequenced with an Illumina NovaSeq 6000 sequencer. Sequencing reads were mapped to GENCODE mouse transcripts reference (release M22, GRCm38.p6) and transcription levels were quantified using salmon. Subsequently, differential expression analysis was carried out using DEseq2 (version: 1.22.2). Benjamini-Hochberg correction for multiple testing was applied to correct p-value of each gene as false discovery rate (FDR). FDR < 0.05 was used as a threshold for differentially-expressed genes (DEGs). Lists of downregulated DEGs and upregulated DEGs were separately examined for statistical enrichment of gene ontology (GO) terms and biological pathways in Toppgene (https://toppgene.cchmc.org). RNA-seq datasets were deposited in Gene Expression Omnibus (GEO) with the accession number GSE222542.

### Statistical analysis

Data are presented as mean ± standard error of the mean (SEM). Statistical analysis was performed using GraphPad Prism 9 software, with Welch’s t test used for comparisons among groups as indicated. P-values less than 0.05 were considered significant and reported as *p < 0.05, **p < 0.01, ***p < 0.001, ****p < 0.0001.

## Supporting information

S1 Fig Filamin C is essential for mammalian heart development. **Related to Fig 1.** (**A**) *Flnc* in situ hybridization images of wild-type mouse embryos from embryonic day (E) 9.5 to E11.5. Black arrows indicate *Flnc* expression in somites. V, ventricle; A, atrium. Scale bar, 1 mm (overview); 0.5 mm (magnified view). (**B**) Representative immunofluorescence (IF) images of control and *Flnc*^*gKO*^ hearts at E9.5 using antibodies against filamin C and α-actinin (cardiomyocyte marker). Scale bar, 0.1 mm. (**C**-**D**) Wholemount images of control and *Flnc* cardiomyocyte-specific knockout embryos with *Xmlc2*^*Cre*^ (**C**) or *cTnT*^*Cre*^ (**D**) at E10.5 and E11.5. Yellow arrows indicate pericardial effusion. Scale bar, 1 mm.
(PDF)

S2 Fig Filamin C maintains the integrity of myocardial wall. **Related to Fig 2.** (**A**) Representative immunofluorescence (IF) images of control and *Flnc*^*gKO*^ hearts at E8.5 using an antibody against cardiac troponin T (cTnT). Scale bar, 0.1 mm. (**B**-**C**) Quantification of cardiomyocyte proliferation rate using phospho-histone H3 (pHH3) IF (**B**) and apoptosis rate using cleaved caspase 3 (cCSP3) IF (**C**) in control and *Flnc*^*gKO*^ hearts from E8.5 to E10.5. Cardiomyocytes were marked with an antibody against cardiac troponin T. n = 3–4 embryos per group; n = 4–6 sections per embryo. n.s., not significant; ****p<0.0001; **p<0.01. (Welch’s t-test) **(D)** Representative immunofluorescence (IF) images of control and *Flnc*^*gKO*^ hearts at E9.5 using antibodies against α-actinin (Z-line) and myomesin (M-line). Scale bar, 10 μm.
(PDF)

S1 Table List of differentially expressed genes (DEGs) in E9.5 *Flnc^gKO^* hearts compared with littermate controls. (XLSX)

S2 Table Numerical data underlying Figs 1C, 3A, 3B, S2B and S2C. (XLSX)
