# Supplementary material for: Filamin C is Essential for mammalian myocardial integrity
Source: PLoS Genet. 2023 Jan 27;19(1):e1010630. doi: 10.1371/journal.pgen.1010630 (PMC9907827; doi:10.1371/journal.pgen.1010630)
Supplement: S1 Fig — Related to Fig 1. (A) Flnc in situ hybridization images of wild-type mouse embryos from embryonic day (E) 9.5 to E11.5. Black arrows indicate Flnc expression in somites. V, ventricle; A, atrium. Scale bar, 1 mm (overview); 0.5 mm (magnified view). (B) Representative immunofluorescence (IF) images of control and FlncgKO hearts at E9.5 using antibodies against filamin C and α-actinin (cardiomyocyte marker). Scale bar, 0.1 mm. (C-D) Wholemount images of control and Flnc cardiomyocyte-specific knockout embryos with Xmlc2Cre (C) or cTnTCre (D) at E10.5 and E11.5. Yellow arrows indicate pericardial effusion. Scale bar, 1 mm. (PDF) [file pgen.1010630.s001.pdf]

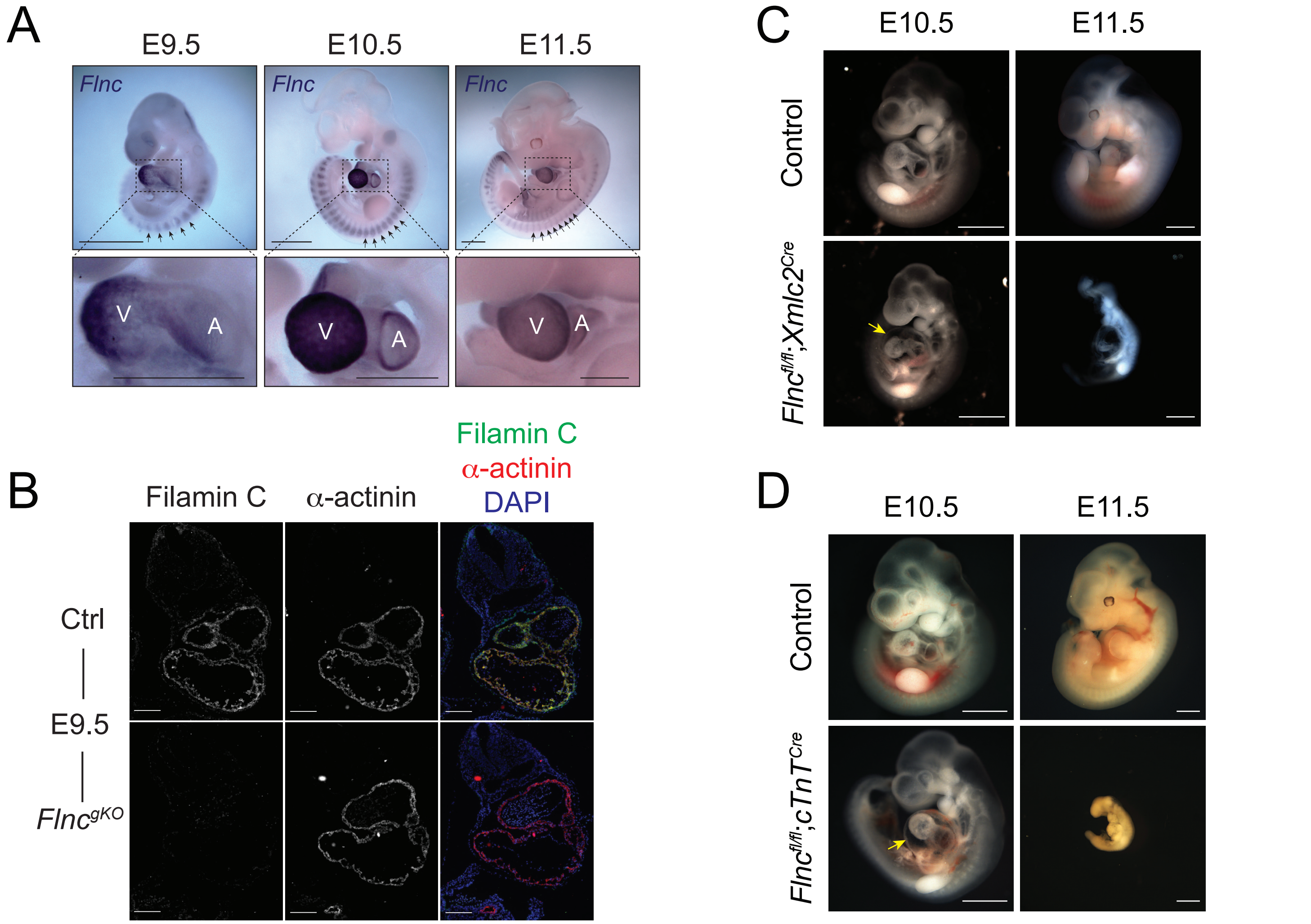

S1 Fig. Filamin C is essential for mammalian heart development. Related to Fig 1. (**A**) *Flnc* in situ hybridization images of wild-type mouse embryos from embryonic day (E) 9.5 to E11.5. Black arrows indicate *Flnc* expression in somites. V, ventricle; A, atrium. Scale bar, 1 mm (overview); 0.5 mm (magnified view). (**B**) Representative immunofluorescence (IF) images of control and *Flnc*^{gKO} hearts at E9.5 using antibodies against filamin C and α-actinin (cardiomyocyte marker). Scale bar, 0.1 mm. (**C-D**) Wholemount images of control and *Flnc* cardiomyocyte-specific knockout embryos with *Xmlc2*^{Cre} (**C**) or *cTnT*^{Cre} (**D**) at E10.5 and E11.5. Yellow arrows indicate pericardial effusion. Scale bar, 1 mm.
