# Supplementary material for: Filamin C is Essential for mammalian myocardial integrity
Source: PLoS Genet. 2023 Jan 27;19(1):e1010630. doi: 10.1371/journal.pgen.1010630 (PMC9907827; doi:10.1371/journal.pgen.1010630)
Supplement: S2 Fig — Related to Fig 2. (A) Representative immunofluorescence (IF) images of control and FlncgKO hearts at E8.5 using an antibody against cardiac troponin T (cTnT). Scale bar, 0.1 mm. (B-C) Quantification of cardiomyocyte proliferation rate using phospho-histone H3 (pHH3) IF (B) and apoptosis rate using cleaved caspase 3 (cCSP3) IF (C) in control and FlncgKO hearts from E8.5 to E10.5. Cardiomyocytes were marked with an antibody against cardiac troponin T. n = 3–4 embryos per group; n = 4–6 sections per embryo. n.s., not significant; ****p<0.0001; **p<0.01. (Welch’s t-test) (D) Representative immunofluorescence (IF) images of control and FlncgKO hearts at E9.5 using antibodies against α-actinin (Z-line) and myomesin (M-line). Scale bar, 10 μm. (PDF) [file pgen.1010630.s002.pdf]

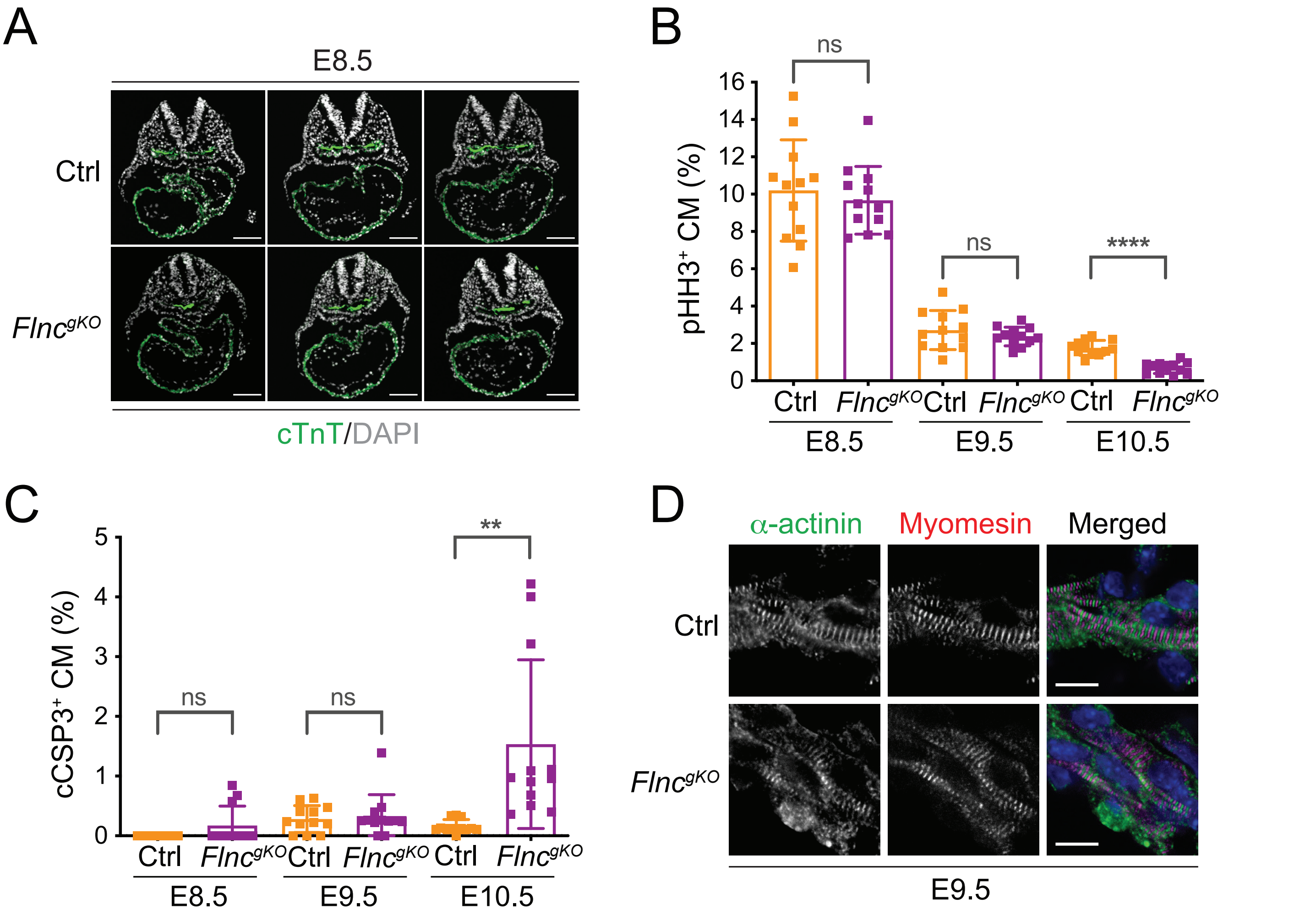

S2 Fig. Filamin C maintains the integrity of myocardial wall. Related to Fig 2. (**A**) Representative immunofluorescence (IF) images of control and *Flnc*^{gKO} hearts at E8.5 using an antibody against cardiac troponin T (cTnT). Scale bar, 0.1 mm. (**B-C**) Quantification of cardiomyocyte proliferation rate using phospho-histone H3 (pHH3) IF (**B**) and apoptosis rate using cleaved caspase 3 (cCSP3) IF (**C**) in control and *Flnc*^{gKO} hearts from E8.5 to E10.5. Cardiomyocytes were marked with an antibody against cardiac troponin T. n=3-4 embryos per group; n=4-6 sections per embryo. n.s., not significant; ****p<0.0001; **p<0.01. (Welch's t-test) (**D**) Representative immunofluorescence (IF) images of control and *Flnc*^{gKO} hearts at E9.5 using antibodies against α-actinin (Z-line) and myomesin (M-line). Scale bar, 10 μm.
